# Social media addiction and personality dimensions among Tunisian medical students

**DOI:** 10.3389/fpsyt.2024.1471425

**Published:** 2024-09-16

**Authors:** Fatma Guermazi, Wissal Abid, Imen Baati, Farah Cherif, Emna Mziou, Dorra Mnif, Ines Feki, Rim Masmoudi, Jawaher Masmoudi

**Affiliations:** ^1^ Psychiatry “A” Department, Hedi Chaker University Hospital, Sfax, Tunisia; ^2^ Community Health B Departement, Faculty of Medicine of Sfax, University of Sfax, Sfax, Tunisia; ^3^ Hospital Hygiene Department, Habib Bourguiba University Hospital, Sfax, Tunisia

**Keywords:** social media, addiction, Big Five personality, self-esteem, medical students

## Abstract

**Purpose:**

Social media (SM) has become a common activity for today’s young people. It is sometimes overused and potentially results in SM addiction. This study aims to assess SM addiction and its associated factors in medical students and to examine its relationship with dimensions of personality global self-esteem, and social self-esteem.

**Materials and methods:**

We carried out a cross-sectional study among a sample of medical students in the region of Sfax in Tunisia. *Social Media Addiction Scale-Student Form (SMAS-SF)*, *Big Five Inventory*, *Rosenberg Self-Esteem Scale*, and *Social Self-Esteem Inventory* were used to collect data.

**Results:**

Overall, 116 medical students were included in the survey. The median age of the participants was 26 years, and 91 students (78.4%) were female. Almost half of them (55.20%) were enrolled in the third cycle. The most widely used SM was Facebook (98.3%). Students with the highest *SMAS-SF* scores had a significantly younger age of first use (p=0.011, r=-0.235), spent more time on their favorite SM (p=0.005, r=0.260), and performed more activities on SM, namely: making comments (p=0.005), browsing SM profiles (p=0.018), and posting videos (p=0.007) or pictures (p=0.002). The need to establish an identity was significantly associated with higher *SMAS-SF* scores (p=0.011). We also found that neuroticism and a low level of conscientiousness were linked to high *SMAS-SF* scores (p=0.006, r=0.252 and p=0.050, r=-0.183, respectively). Moreover, high *SMAS-SF* scores were significantly related to lower *global* and *social self-esteem* scores (p=0.015, r=-0.226 and p=0.032, r=-0.199, respectively).

**Conclusion:**

Our results highlight the critical need to take into consideration the evaluation and intervention of self-esteem and personality dimensional issues to target interventions for SM addiction among medical students.

## 1 Introduction

Nowadays, the Internet is increasingly involved in a wide range of everyday activities. Social media (SM) has revolutionized the Internet, and its use is increasing worldwide, particularly among young people, who are showing a keen interest in it (1, 2). According to a report in 2023, more than half of the world’s population actively uses SM (3). These web-based platforms have become very popular tools that enable users to build interactive online communities and disseminate all types of information (4, 5); however, these platforms could also be used to enhance educational interactions and learning in students (6–8). For medical students, SM can provide platforms facilitating efficient interactions and connections among health professionals in clinical practice, professional networks, education, and training (9).

Nevertheless, extensive use of SM may be problematic because it can lead to behavioral addiction. SM addiction is a relatively new phenomenon that has become a serious public health concern due to its significant impairment of users’ functioning in important life domains, such as interpersonal relationships, work or academic performance, and physical and/or psychological health and well-being, especially among younger people (10–17).

The prevalence rates and scores of SM addiction reported in the literature vary greatly between the studies. In 32 countries, the prevalence of SM addiction was recently evaluated by a meta-analysis, which revealed a pooled prevalence of 24% (18).

Studies among university students, who tend to be the main users of the virtual world and SM, reveal high rates of addictive SM behavior in Africa (19), with prevalence estimates ranging from 8% in Ethiopia (20) to 13% in Nigeria (21).

Medical students could be particularly vulnerable to problematic SM use. In fact, many are found to be suffering from various psychological distress due to their challenging academic obligations, and the use of virtual SM platforms may help them tremendously in coping with stressors and induced negative emotions (22, 23). Regrettably, the addiction to SM in these future physicians could interfere with both their ability to perform their daily tasks and with their learning process, which may affect their future performance in the medical profession.

Despite this empirical evidence of the scale and seriousness of this phenomenon, there is a lack of studies examining the underlying psychopathology that predicts the development of such an addiction. In fact, like other behavioral addictions, the phenomenon of SM addiction is influenced by various biopsychosocial factors (24), some of which have been advanced under psychological theories, such as social comparison theory, uses and gratifications theory, or emotion regulation theory (25–27).

Among these factors, personality plays a significant role as an individual difference that shapes the motivations and gratifications related to SM use (28). Numerous studies have sought to understand the types of people who are drawn to SM by investigating the relationship between SM use and personality (29). Many studies based on the Big-Five model of personality have shown that certain dimensions are particularly involved in SM use and addiction (10, 28, 30–32). However, these studies have often yielded mixed results. In addition, students are classically described in the literature as a population with low self-esteem. This concept of self-esteem, which refers to an individual’s evaluation or judgment of himself or herself, may lead us to question its role in the development of such an addiction to the virtual world (15, 33, 34).

Given all these considerations, the current study aimed to assess SM addiction and its environmental determinants among a sample of Tunisian medical students and to examine its relationship with dimensions of personality, global self-esteem, and social self-esteem.

## 2 Materials and methods

### 2.1 Sample and procedure

This is a cross-sectional, descriptive, and analytical web-based survey, conducted between November 2022 and January 2023. The questionnaire was published via medical student-related Facebook discussion groups. Participants were recruited based on a convenience sampling of medical students enrolled at the Faculty of Medicine of Sfax, who met the inclusion criteria.

The research population consisted of medical students of the Faculty of Medicine of Sfax, in Tunisia. The inclusion criteria included students with access to the Internet who were voluntarily willing to participate in the study and to complete the questionnaire fully.

The minimum necessary sample size was 174 students and was calculated based on data from (18) using the following formula:


$$n=\frac{NZ^{2}pq}{\left(e^{2}\left(N-1\right)+Z^{2}pq\right)\;}$$

n=sample size; N=population size; Z=the statistic corresponding to level of confidence; p=proportion of SM addiction; q=1-P; e=precision. The estimation was also based on a 5% margin of error, and a 95% confidence interval.

All the procedures performed in this study were in accordance with the ethical standards of the “Research Ethics Committee, Faculty of Medicine, University of Sfax, Tunisia” and with the 1964 Helsinki Declaration and its later amendments or comparable ethical standards. This research was approved by the above-mentioned committee (approval number 20/24).

### 2.2 Data collection and measurements

Data were collected using an anonymous self-report questionnaire that was edited by free GOOGLE FORMS software and sent via a URL link with an information note presenting the scientific aim of the study, with a focus on ensuring the confidentiality and anonymity of responses. Throughout the study period, the questionnaire was published via medical student-related Facebook discussion groups.

We used a survey form to collect sociodemographic and academic characteristics, data related to lifestyle habits, personal and family clinical history, and characteristics of SM use. We also used four psychometric scales: *Social Media Addiction Scale-Student Form (SMAS-SF), Big Five Inventory (BFI-10), Rosenberg Self-Esteem Scale (RSES), and Social Self-Esteem Inventory (SSEI).*

#### 2.2.1 Social Media Addiction Scale-Student Form (SMAS-SF)

It is a tool validated in English by Sahin in 2018 to assess problematic SM use (2). It assesses addiction to social media in general among students. This is a 5-point Likert-type scale, ranging from 1 (disagree strongly) to 5 (agree strongly) and consisting of 29 items grouped under 4 sub-dimensions (virtual tolerance, virtual communication, virtual problem, and virtual information). The highest point that can be scored on the scale is 145, and the lowest one is 29. Higher scores indicate that the individual perceives himself as a *social media addict*. The Cronbach’s alpha coefficient was found to be 0.93 for the whole scale (2).

#### 2.2.2 Big Five Inventory (BFI-10)

It is a self-administered questionnaire created by John et al. in 1990 to provide clinicians and researchers with a quick and reliable tool for measuring individual differences along Goldberg’s five major personality dimensions (35, 36). We used the short, validated version of the Big Five Inventory (BFI-10) developed by Gosling et al. in 2003 (37). The inventory comprises the Big Five personality dimensions which are neuroticism, extraversion, conscientiousness, agreeableness, and openness to experience. Each dimension has two items on a 5-point Likert scale, ranging from 1 (strongly disapproves) to 5 (strongly approves). The minimum and maximum scores for each dimension are 2 and 10, respectively. The highest-scored personality dimension is identified as the participant’s dominant personality trait. The Cronbach’s alpha coefficient was 0.73.

#### 2.2.3 Rosenberg Self-Esteem Scale (RSES)

This scale is the most widely used in psychological research to measure global self-esteem. It was developed by sociologist Morris Rosenberg (38). We used the French version validated by Vallières and Vallerand et al. (39). The scale consists of 10 items (5 are positive statements and 5 are negative statements). The *RSES* is usually scored as a 4-point Likert scale ranging from “Strongly Agree” to “Strongly Disagree.” The scale ranges between 10 and 40 and guides us to 3 possible levels of self-esteem: Low level (10–25), medium level (26–29), and high level (30–40) (40). The Cronbach’s alpha coefficient was 0.70.

#### 2.2.4 Social Self-Esteem Inventory (SSEI)

This inventory was translated into French by Gauthier et al. (41) and validated by Bouvard et al. in 1999 (42). It is a uni-dimensional self-questionnaire composed of 30 items that measure self-esteem in social situations. Participants rate each situation on a 6-point Likert scale (from 1: completely different from me to 6: exactly like me). The total score ranges from 30 to 180. The higher the score, the greater the individual’s social self-esteem (17). The Cronbach’s alpha coefficient was 0.96.

### 2.3 Statistical analysis

The collected data were analyzed using the Statistical Package for Social Sciences (SPSS) software, version 22. We used the Kolmogorov-Smirnov test to determine if the variables were normally distributed. Quantitative variables were presented as mean and standard deviation (SD) or median and interquartile range (IQR), according to the variable distribution. Qualitative variables were represented as numbers and percentages. Statistical comparisons of the analytical study were carried out using Pearson’s r-correlation test for quantitative variables and the power of the correlation was estimated using the rho. For qualitative variables it was carried out using Mann–Whitney U-Test. All tests were two-tailed at a 95% confidence interval, and the *p-value* was considered significant if <0.05.

## 3 Results

### 3.1 Complete-response rate

During the study period, 150 students were voluntarily enrolled. Thirty-four participants did not answer certain questions and were consequently excluded from the study. Thus, a total of 116 students, who filled out the survey completely, were retained in this study (complete-response rate of 77.33%).

### 3.2 Sociodemographic, academic, and clinical history characteristics

Of the 116 medical students participating in the study, 91 students (78.4%) were female. The majority (77.6%) were between 21 and 30 years of age. Almost half of them (55.20%) were enrolled in the third cycle. Concerning their clinical history, 15.5% of respondents reported having psychiatric problems, and 6% were receiving psychotropic treatment. Smoking and alcohol use were reported by 9.5% and 1.7% of the participants, respectively (**Table 1** **).**

**Table 1:** Demographic, academic, and clinical history characteristics of respondents.

Variable	n	%
Gender		
Male Female	25 91	21.6% 78.4%
Age groups		
Age ≤ 20 years 21 years ≤ Age ≤ 30 years Age > 30 years	10 90 16	8.6% 77.6% 13.8%
Living area		
Rural Urban/semi-urban	3 113	2.6% 97.4%
Marital status		
Single or divorced or widower Married	94 22	81% 19%
Educational level		
First cycle Second cycle Third cycle	34 18 64	29.3% 15.5% 55.2%
University repeat		
Yes No	19 97	16.38% 83.62%
Leisure activity		
Yes No	36 80	31.03% 68.97%
Psychoactive Substance Use		
Tobacco Alcohol Cannabis	11 2 1	9.5% 1.7% 0.9%
Personal history of somatic disease		
Yes No	31 85	26.7% 73.3%
Psychiatric personal history		
Anxiety disorder Bipolar disorder Schizophrenia Recurrent depression Others	8 4 1 3 2	6.9% 3.4% 0.9% 2.6% 1.7%

n, number; %, percentage.

### 3.3 Social media-specific data

**Figure 1:**
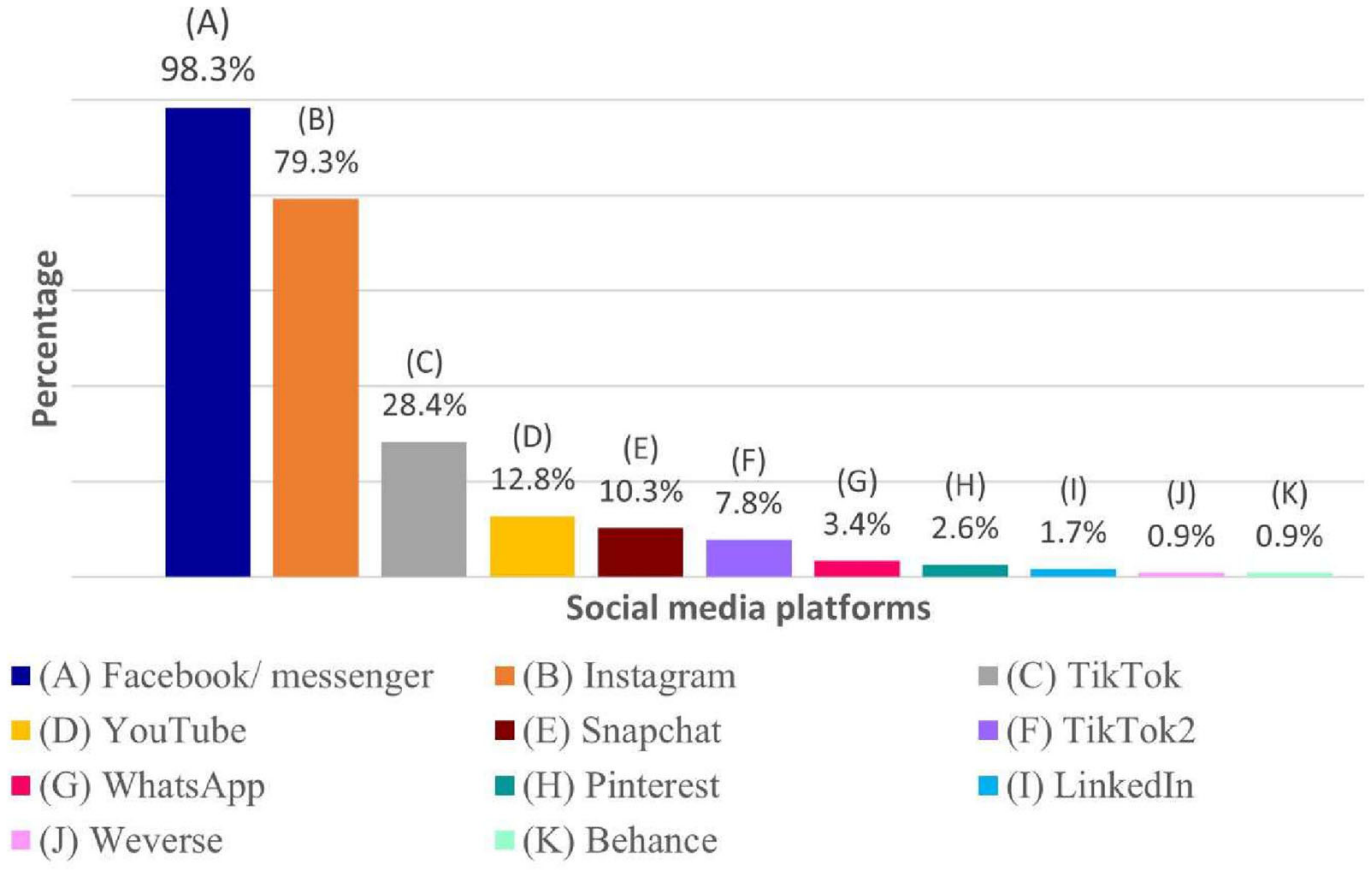
Breakdown of respondents by most frequently used social media platforms. Social media platforms: **(A)** Facebook/Messenger **(B)** Instagram **(C)** TikTok **(D)** YouTube **(E)** Snapchat **(F)** TikTok2 **(G)** WhatsApp **(H)** Pinterest **(I)** Linked In **(J)** Weverse **(K)** Behance.

In our study, Facebook (98.3%) was the most used SM, followed by Instagram (79.3%) and TikTok (28.4%) (**Figure 1** **).** The other SM platforms remained below 50%.

The description of SM-specific data among the participants is shown in **Table 2**. The table shows that the median age of the first use of SM was 14.5 years (IQR=[12-17]). The median number of hours spent per day on a favorite SM was 2 (IQR= [1-3]). The median number of followers/friends or subscribers was 600 (IQR= [300-1100]). When asked which actions they carried out most often on SM, students highlighted sharing private messages in 76.7% of cases. The most frequent motivation to connect to SM reported by students was a need to keep in touch with friends (82.8%).

**Table 2:** Data related to the use of SM associated with *SMAS-SF* scores.

Variable		n (%)	*SMAS-SF* scores Median [IQR]	*p-value*
**Age of first use**		**-**	14.5 [12-17]	**0.011** **(rho=**-**0.235)**
**Number of SM used**		**-**	3 [2-3]	0.069
**Number of hours per day spent on all SM**		**-**	4 [2-5.7]	**0.008** **(rho=0.244)**
**Number of hours spent on favorite SM**		**-**	2 [1-3]	**0.005** **(rho=0.260)**
**Number of subscribers**		**-**	600 [300-1100]	0.098
**SM actions**	Share private messages	**89** (**76**.**7**)	–	0.094
	Share links	46 (39.7)	–	0.110
	Make comments	25 (21.6)	–	**0.005**
	Browse other users’ profiles	24 (20.7)	–	**0.018**
	Post pictures	34 (29.3)	–	**0.002**
	Post videos	15 (12.9)	–	**0.007**
	Watch videos/real	8 (6.9)	–	0.651
	Visit online pages	4 (3.4)	–	0.051
**Motivations for SM connection**	Keep in touch with friends	**96 (82.8)**	–	0.192
	Need for information	88 (75.9)	–	0.892
	Listening to music	51 (44)	–	0.898
	Need to express a feeling	25 (21.6)	–	0.066
	Need to establish an identity	19 (16.4)	–	**0.011**
	Need to play games	12 (10.3)	–	0.079
	Need to earn others’ respect	8 (6.9)	–	0.827

SMAS-SF, Social Media Addiction Scale-Student Form; SM, social media; n, number; %, percentage; IQR, Interquartile range. Bold values represent statistically significant results.

According to *SMAS-SF*, the scores ranged from 38 to 141, with a median score of 74 and (IQR= [64-587]).

### 3.4 Psychometric assessment of personality dimensions

The description of the participants’ scores on the Big Five personality dimensions is presented in **Table 3**.

**Table 3:** Summary of Big Five personality dimension scores.

Big Five personality dimension	Median	IQR
Dimension “extraversion”	6	[5-7]
Dimension “agreeableness”	7	[6-8]
Dimension “conscientiousness”	7	[6-8]
Dimension “neuroticism”	6	[5-8]
Dimension “openness”	7	[6-8]

IQR, Interquartile range.

### 3.5 Psychometric assessment of self-esteem

The mean score of *RSES* in the study sample was 30.4 ± 5.09. Of the students surveyed, 12.1% had low global self-esteem (**Table 4**).

**Table 4:** Distribution of students based on their levels of *Rosenberg Self-Esteem Scale*.

*RSES* levels	n	%
Low	14	12.1
Medium	50	43.1
High	52	44.8
Total	116	100

n, number; RSES, Rosenberg Self-Esteem Scale.

According to the *SSEI scale*, the mean score was 122.03 ± 23.25.

### 3.6 Factors associated with social media addiction

No significant associations were observed between the *SMAS-SF* score and sociodemographic and academic data. Taking psychotropic medication was significantly associated with higher *SMAS-SF* scores (p=0.028) (**Table 5**).

**Table 5:** Sociodemographic, academic and clinical history factors associated with *SMAS-SF* scores.

Variable	n		*SMAS-SF* scores median [IQR]	p-value
**Age > 20 years**	Yes	106	74 [63.75-86.25]	0.096
	No	10	81[74-108.5]	
**Gender**	Male	25	84 [66-98]	0.057
	Female	91	74 [64-85]	
**Marital status**	Married	22	71 [53.75-87.25]	0.223
	Single-Divorced –widower	94	75 [66-87.25]	
**Presence of child**	Yes	16	71 [51.75-81.5]	0.149
	No	100	75 [66-87.75]	
**Living area**	Urban/semi-urban	113	74 [64-87.5]	0.676
	Rural	3	72 [68-78]	
**Educational level**	First cycle	52	77.5 [67.5-89]	0.556
	Second and third cycles	64	74 [63.75-85.25]	
**University repeat**	Yes	19	73 [56-86]	0.641
	No	97	75 [65-87.5]	
**Leisure activity**	Yes	36	75.5 [66.5-84]	0.877
	No	80	74 [64-88.75]	
**Psychoactive Substance Use**	Yes	14	55.5 [28.5-54.75]	0.171
	No	102	74 [65.5-87.5]	
**Personal history of somatic disease**	Yes	31	75 [66-94]	0.447
	No	85	74 [64-86.5]	
**Psychiatric personal history**	Yes	18	76 [69.5-89.5]	0.504
	No	98	74 [63-87]	
**Psychotropic treatment**	Yes	6	101.5 [72.75-115.25]	**0.028**
	No	110	74 [63-75.86]	

n, number; SMAS-SF, Social Media Addiction Scale-Student Form; IQR, Interquartile range. Bold values represent statistically significant results.

As shown in **Table 2**, participants with the highest *SMAS-SF* scores had a significantly younger age of first use (p=0.011, rho=-0.235). Students who spent more time on their favorite SM were more likely to develop an SM addiction (p=0.005, rho=0.260). We found that some activities performed on SM were significantly associated with higher *SMAS-SF* scores, namely: making comments (p=0.005), browsing SM profiles (p=0.018), and posting videos (p=0.007) or pictures (p=0.002). The lower panel of **Table 2** similarly **s**hows higher scores of *SMAS-SF* among students whose motivation to use SM was the need to establish an identity (p=0.011).

By examining the relationship between personality dimensions and SM addiction, it appeared that neuroticism and a low level of conscientiousness were linked to high *SMAS-SF* scores (p=0.006, rho=0.252 and p=0.050, rho=-0.183, respectively) (**Table 6**).

**Table 6:** Results of the Nonparametric Test for the association of scores of *SMAS-SF* and personality dimensions, Global and social self-esteem scores.

	*SMAS-SF* scores	
	*p-value*	*Rho*
Big Five personality dimension scores:		
**• Dimension “extraversion”** **• Dimension “agreeableness”** **• Dimension “conscientiousness”** **• Dimension “neuroticism”** **• Dimension “openness”**	0.397 0.997 **0.050** **0.006** 0.898	**-** **-** -**0.183** **0.252** **-**
**Global self-esteem scores**	**0.015**	-**0.226**
**Social self-esteem scores**	**0.032**	-**0.199**

SMAS-SF, Social Media Addiction Scale-Student Form. Bold values represent statistically significant results.

We also found that high *SMAS-SF* scores were significantly related to lower global and social self-esteem scores (p=0.015, rho=-0.226 and p=0.032, rho=-0.199) (**Table 6**).

## 4 Discussion

To the best of our knowledge, this is the first study to use the *SMAS-SF* to assess SM dependence among a sample of Tunisian medical students, in the region of Sfax. According to this scale, a median score of 74 can be considered a moderate level of SM addiction.

### 4.1 Factors associated with social media addiction

In our study, taking psychotropic medication was significantly associated with higher *SMAS-SF* scores. A wealth of evidence suggests psychological distress is associated with higher levels of SM use (43, 44). The authors supported the idea that when using SM, people might engage in “harmful” comparisons in which they make erroneous inferences about the lives of other users. These comparisons could explain why SM use often leads to depressive symptoms that need treatment (45).

Our results showed that *SMAS-SF* scores were significantly higher among students with a younger age of first use. Several academic publications agree that SM use is highly normative among today’s adolescents and young individuals, who represent a unique population, frequently engaged in online activities, and are the first generation to grow up in a highly digitalized society (43, 46). Indeed, youngsters have more free time than older people, do not yet have full-time professional preoccupations, and are more interested in forging new relationships through social networking (43, 46, 47).

The most widely used SM among our participants was Facebook (98.3%), followed by Instagram and TikTok. These results are comparable to those found in another recent Tunisian research (48). Indeed, the birth of Facebook in 2004 left its mark on the internet, offering a new concept that continues to spread throughout the world in general, and consequently in Tunisia. Considered the archetypal online SM, it gained widespread popularity in a very short time. It has accumulated the highest number of users in the world (49). Regarding Instagram, recent statistics show that it is a relatively new SM that is gradually becoming one of the most popular platforms. This SM offers users an immediate means of capturing and sharing their personal experiences (50).

Furthermore, our findings show that participants who have the highest *SMAS-SF* scores spent more time connecting on all SM or on their favorite SM, and performed more SM activities, namely, making comments, browsing SM profiles, and posting videos and/or photos. These results are comparable to those reported in the literature, which show reported positive associations between daily internet use and SM addiction (51, 52). In this respect, Sun et al. have shown that signs of SM addiction can include logging on to SM for more than an hour a day and being curious to see the profiles of old friends (53). The study by Kircaburun et al. showed that introverts spend a lot of time on SM and use this time mainly to consult other people’s profiles without any interaction to avoid real-life confrontations and social interactions (52). On the other hand, it has been found that some activities, such as reading comments and sharing videos on YouTube, are associated with the gratification of entertainment (54). This may partly explain the excessive use of this SM to seek this effect.

In terms of motivations for connecting to SM, the “need to establish an identity” was associated with the highest *SMAS-SF* scores. Indeed, it has been shown that virtual contexts can be a means of escaping personal difficulties or compensating for a lack of social skills that would make face-to-face communication difficult (55). Users of SM display an idealized identity by presenting themselves as they wish in a positive way, and by controlling the information and elements integrated into their profiles (56). SM users construct their ideal selves, or “hoped-for possible selves”, as part of a mediated self-presentation (57). Research indicates that young people’s SM behavior involves self-exploration, which is potentially associated with the crucial task of identity development (58).

### 4.2 Links between social media addiction and personality dimensions

In our study, neuroticism and a low level of conscientiousness were linked to the highest SMAS-SF scores.

According to our findings, previous research identified the neuroticism dimension as a predictor of SM addiction (17, 28–30, 59). People with high scores in neuroticism used the Internet frequently, spent more time on Facebook, and sent more instant messages (60). Indeed, neurotics are characterized by anxiety and insecurity in their relationships, leading them to feel powerless (61). This would lead to excessive use of SM in order to seek support and companionship to cope with this emotional distress. In addition, neurotics are more likely to have problematic SM use because they want to relieve loneliness and increase their self-esteem by joining social groups and developing a sense of group belonging, albeit virtual (59, 62).

Conscientiousness refers to impulse control, planning, and organization. It has been well established, in prior research, that this personality dimension protects against problematic SM use (27, 60, 63). The authors explain that conscientious people give less priority to virtual activities in order to fulfill other obligations and meet deadlines for real tasks (17). These people tend to cultivate their online and offline contacts more without the necessity of sharing too much personal information publicly. They upload significantly fewer pictures than those scoring low on this personality trait (62).

Regarding extraversion, its relationship with SM addiction has been controversial. While some authors showed that extraversion predicts SM addiction (64), others, in line with our findings, found no relationship between social media addiction and extraversion (31). Extroverts have more online friends and are more self-disclosing, posting more selfies and statuses frequently (65). However, they do not use online socializing as a substitute for real-life social interaction. Thus, extroverts use SM primarily for social enhancement (62, 66).

Several psychological theories have been advanced to explain the influence of these personality traits on the development of SM addiction.

A large body of literature supports the idea that young people with frequent and unbalanced negative affect have been found to be more likely to engage in addictive behavior as a means of coping with high levels of stress. Such individuals experience difficulties in developing healthy relationships, due to their problematic management of emotional states, and consequent mood disorders. They may consider the internet an arena in which they can achieve greater self-control and engage in better communication with others (26, 27). Quaglieri et al. (27) found that individuals with high levels of neuroticism and low levels of conscientiousness showed poor emotional coping skills and experienced more depression, anxiety, and emotional dysregulation prior to the onset of their addiction, which likely emerged as an attempt to ameliorate these negative symptoms. These findings seem to be in line with the “compensatory Internet use” model developed by Kardefelt-Winther (67), which proposed Internet addiction as a coping strategy and presented it from the perspective of compensation rather than compulsive behavior. Individuals who experience negative life situations are more likely to overuse online activities in the search for instant gratification in order to ameliorate their dysphoric mood problems (27, 63).

Furthermore, several studies have linked the “fear of missing out” to the overuse of SM (68, 69), which may be considered ideal for satisfying the “desire to stay continuously connected to what others are doing”. The continuous exposure to this information could lead individuals to believe that “others” are having a better life and that one is missing out on something. This could place the individual in a reinforcement spiral of behavior, making it easier to exacerbate the anxiety and loss of control that characterize neurotics and less conscientious people (27).

Other studies have proposed a mediating role of social comparison theory in the relationship between SM addiction and personality (25, 63). This theory suggests that individuals have a natural tendency to evaluate their abilities by comparing themselves to others (70). However, when using SM, people may be exposed to idealized and unrealistic profiles. Therefore, those with certain fragile personality traits, particularly high levels of neuroticism and low levels of conscientiousness, may end up feeling envy and psychological distress (25). In fact, negative social comparisons on SM platforms can be particularly detrimental to perceptions about their selves. Given this, Piko et al. (63) suggested that fear of negative evaluation can play a decisive role in SM addiction.

### 4.3 Links between social media addiction and self-esteem

In our study, high *SMAS-SF* scores were significantly associated with lower global and social self-esteem scores.

Most studies confirmed the negative relationship between self-esteem and SM addictive use (15, 32, 34, 62, 71, 72).

A person’s self-esteem is tied most to the amount of active engagement of their own SM and also to the engagement of others with their SM. The low levels of social self-esteem in participants with problematic SM use could be explained by the low sense of popularity induced by social comparison. Some authors have highlighted the deleterious effect of intensified social self-comparison among SM users on others perceived as more valued than themselves. These biases of comparison could contribute to increasing young people’s vulnerability. Indeed, the sociometer theory, a prominent theory of self-esteem, suggests that a person’s self-worth is primarily derived from the feedback they receive from others’ virtual profiles (10, 17, 56, 70, 73).

On the other hand, the recent rise of SM usage has been considered a safer societal interaction tool that has the potential to relieve the social anxiety that people with low self-esteem often experience in face-to-face communications. Indeed, the anonymity or selectiveness of self-presentation on SM allows them to feel more relaxed and confident in order to express themselves, build their desired persona online, withdraw from the negative evaluations and stress of interpersonal relationships encountered in real life, and escape negative feelings of self-deprecation (10, 15, 34, 74, 75). Likewise, if someone is mostly involved in negative interactions on these sites, an adverse influence on his or her social self-esteem seems plausible. By contrast, participants can usually more easily eliminate undesirable encounters or feedback and focus entirely on positive experiences, thereby enhancing their social self-esteem (76).

Moreover, research suggests that fear of missing out which is a psychological state in which people become anxious that others within their social spheres are leading much more interesting and socially desirable lives, mediates the relationship between increased SM use and decreased self-esteem (69).

## 5 Limitations

To the best of our knowledge, our study is the first to study SM addiction and its relationship with dimensions of personality and global and social self-esteem among medical students in the region of Sfax in Tunisia. However, some limitations of this study should be taken into account when interpreting the results. The first is the limited sample size, which was unfortunately smaller than theanticipated size. This may be due to the recruitment procedure based on convenience sampling. Second, our sample may not be representative of the whole population of Tunisian medical students. Further studies with a more representative sample of medical students, in other cities in Tunisia would be interesting to support the findings of our study. Third, including students with psychiatric and mental illnesses (e.g., schizophrenia, bipolar disorder, etc.) may bias the results. Finally, the cross-sectional nature of the study does not enable us to follow the longitudinal changes. Cohort studies should be conducted to better explore the negative consequences of SM addiction and to help examine causal relationships more fully.

## 6 Conclusion and implications for practice

The findings of our study helped identify a profile of the “social media addict” user. He/She is often a young student receiving psychotropic treatment and exhibiting high levels of neuroticism and low levels of conscientiousness, as well as low overall and social self-esteem.

Because of the profile of these at-risk subjects, it is necessary to address the management of the aforementioned disorders in an integrated and concurrent manner due to their impact on the socio-familial life and academic performance of young students.

The practical implications stemming from this are noteworthy, given the impact on the social-family life and academic performance of young students. In the face of these serious psychological issues, it is recommended to design and implement programs aimed at promoting mental health. Proper planning is required for the prevention of social media addiction, controlling its usage, and reducing the associated risks.

Cognitive-behavioral therapy is also suggested to combat this type of behavioral addiction. Thus, a therapy focused on self-acceptance or self-affirmation could help reduce the need to use social media for satisfying interactions. Acquiring a more optimal mode of relationship through a secure therapeutic relationship could also be an objective of intervention. These therapies also encourage young people considered dependent to engage in other activities, mainly social and non-virtual ones, allowing for personal fulfillment with the aim of personal satisfaction, tolerance, and self-acceptance. Moreover, these therapies target emotional distress and would enable these young individuals to improve their ability to manage their emotions and cope with negative ones. Furthermore, prevention efforts targeting psychologically vulnerable individuals could be expanded to all sectors.

However, further research, preferably utilizing more representative samples, on these understudied relationships is required to yield more conclusive results.

## Data Availability

The raw data supporting the conclusions of this article will be made available by the authors, without undue reservation.
